# Numerical identification of epidemic thresholds for susceptible-infected-recovered model on finite-size networks

**DOI:** 10.1063/1.4922153

**Published:** 2015-06-04

**Authors:** Panpan Shu, Wei Wang, Ming Tang, Younghae Do

**Affiliations:** 1 Web Sciences Center, University of Electronic Science and Technology of China, Chengdu 610054, China; 2 State key Laboratory of Networking and Switching Technology, Beijing University of Posts and Telecommunications, Beijing 100876, China; 3 Department of Mathematics, Kyungpook National University, Daegu 702-701, South Korea

## Abstract

Epidemic threshold has always been a very hot topic for studying epidemic dynamics on complex networks. The previous studies have provided different theoretical predictions of the epidemic threshold for the susceptible-infected-recovered (SIR) model, but the numerical verification of these theoretical predictions is still lacking. Considering that the large fluctuation of the outbreak size occurs near the epidemic threshold, we propose a novel numerical identification method of SIR epidemic threshold by analyzing the peak of the epidemic variability. Extensive experiments on synthetic and real-world networks demonstrate that the variability measure can successfully give the numerical threshold for the SIR model. The heterogeneous mean-field prediction agrees very well with the numerical threshold, except the case that the networks are disassortative, in which the quenched mean-field prediction is relatively close to the numerical threshold. Moreover, the numerical method presented is also suitable for the susceptible-infected-susceptible model. This work helps to verify the theoretical analysis of epidemic threshold and would promote further studies on the phase transition of epidemic dynamics.

Epidemic threshold, which is one of the most important features of the epidemic dynamics, has attracted much attention recently. The existing studies have provided different theoretical predictions for epidemic threshold of the susceptible-infected-recovered (SIR) model on complex networks, while the numerical verification of these theoretical predictions is still lacking. As a result, it is very necessary to develop an effective numerical measure for identifying the SIR epidemic threshold. In this paper, the numerical identification of the SIR epidemic threshold is systematically studied. We present a numerical method by analyzing the peak of the epidemic variability to identify the epidemic threshold. To understand the effectiveness of the variability measure, the distribution of outbreaks sizes is investigated near the epidemic threshold on random regular networks. Based on the analysis of the cutoff hypothesis of the outbreak size distribution, we find that the variability measure can provide an excellent identification of the epidemic threshold. We further use the variability measure to verify the existing theoretical predictions on scale-free and real networks. The results show that the heterogeneous mean-field (HMF) prediction agrees very well with the numerical threshold, except the case that the networks are disassortative, in which the quenched mean-field (QMF) prediction is relatively close to the numerical threshold. The numerical method presented can effectively identify SIR epidemic thresholds on various networks, and could be extended to other dynamical processes such as information diffusion and behavior spreading. This work provides us a deep understanding of epidemic threshold and would promote further studies on phase transition of epidemic dynamics.

## I. INTRODUCTION

Models for disease propagation are at the core of our understanding about epidemic dynamics on complex networks.^1,2^ Two epidemic models of particular importance are the susceptible-infected-susceptible (SIS) and susceptible-infected-recovered (SIR) models.^3^ At each time step, an infected node can transmit a disease to each of its susceptible neighbors with probability *β*. At the same time, the infected nodes become susceptible again in the SIS model or recover in the SIR model with probability *μ*. In the SIS model, a critical value of the effective transmission rate λ=β/μ separates the absorbing phase with only healthy nodes from the active phase with a stationary density of infected nodes. Differently, no steady state is allowed in the SIR model, but a threshold still exists above which the final fraction of recovered nodes is finite.^4^

The traditional theoretical study on the epidemic threshold of the SIS model is based on the heterogeneous mean-field (HMF) theory, which means that all the nodes within a given degree are considered to be statistically equivalent.^5,6^ According to the HMF theory, the epidemic threshold of SIS model^7,8^ is given by $\lambda_{c}^{HMF}=\frac{〈k〉}{〈k^{2}〉}$, where 〈k〉 and $〈k^{2}〉$ are the first and second moments of degree distribution *P*(*k*),^9^ respectively. As the quenched structure of the network and dynamical correlations between the state of adjacent nodes is neglected in the HMF theory,^10^ researchers have proposed an important improvement over the HMF theory—quenched mean-field (QMF) theory. The QMF theory fully preserves the actual quenched structure of the network described as its adjacency matrix, and the epidemic threshold is predicted to be^11–13^

$$\lambda_{c}^{QMF}=\frac{1}{\Lambda_{N}},$$ (1)

where Λ_*N*_ is the maximum eigenvalue of the adjacency matrix of a given network. Considering that the existing theories more or less have some limitations (e.g., the HMF theory neglects the quenched structure of the network; QMF theory ignores dynamical correlations^14^), some numerical methods such as the finite-size scaling analysis,^15^ susceptibility,^16^ and lifetime measure^17^ have been proposed to check the accuracies of different theoretical predictions for the SIS model. Among these existing methods, the relatively common one for a network with finite size *N* is the susceptibility measure

$$\chi =N\frac{〈\rho^{2}〉-{〈\rho 〉}^{2}}{〈\rho 〉},$$ (2)

where *ρ* denotes the outbreak size. In Ref. 18, the susceptibility measure has been shown to be very effective for identifying the SIS epidemic thresholds on various networks.

For another paradigmatic epidemic model, the SIR model, there have been a lot of theoretical studies on its epidemic threshold. The earliest theoretical study on the SIR epidemic threshold is based on the assumption of homogeneous mixing, showing that the SIR epidemic threshold is inversely proportional to the average connectivity 〈k〉.^3^ At the HMF level,^19^ the epidemic threshold of SIR model takes the value

$$\lambda_{c}^{HMF}=\frac{〈k〉}{〈k^{2}〉-〈k〉}.$$ (3)

On networks with power-law scaling $P(k)∼k^{-\gamma }$,^9,20^ where *γ* is the degree exponent, the HMF approach predicts a vanishing threshold for scale-free networks with γ≤3 and the finite threshold for γ>3.^4^ Mapping the SIR model to a bond percolation process,^21^ the epidemic threshold coincides with the result of Eq. (3) for a SIR model with unit infection time (e.g., *μ* = 1), and when the infection times vary among infected nodes (e.g., μ<1), the epidemic threshold is given by^4^

$$\lambda_{c}=\frac{〈k〉}{〈k^{2}〉-2〈k〉}.$$ (4)

According to the QMF theory,^11^ the epidemic threshold of the SIR model has the same expression as Eq. (1). However, the QMF result is even qualitatively not correct, as the QMF predicts a vanishing threshold for power-law distributed networks with γ>3 that is in conflict with the visually numerical results.^22^

Although the numerical threshold of the SIS model has attracted much attention,^15–18^ the systematic study of the numerical identification of the SIR epidemic threshold is still insufficient. It is well known that the outbreak size becomes finite above the threshold *λ_c_*.^21^ However, as the value of *λ* increases, the outbreak size continuously changes from an infinitesimal fraction to a finite fraction in the SIR model,^23^ and thus, it is difficult to determine the value of *λ* at which the outbreak size turns to be finite. To our knowledge, there has no effective numerical method for identifying the SIR epidemic threshold in previous studies. In this work, we perform extensive numerical simulations of the SIR model on networks with finite size, and present a numerical identification method by analyzing the peak of the epidemic variability^24,25^ (i.e., the maximal value of the epidemic variability) to identify the epidemic threshold. The effectiveness of the numerical measure is checked on random regular networks (RRN), where the HMF theory is exact. To get a deep understanding of the validity of the numerical method, we investigate the distribution of outbreaks sizes near the epidemic threshold. The robustness of the variability measure is confirmed by the analysis of cutoff hypothesis of the outbreak size distribution. We further employ the variability measure to verify the theoretical predictions on scale-free networks and real-world networks, where the results indicate that the HMF prediction agrees very well with the numerical threshold, except the case that the networks are disassortative, in which the QMF prediction is relatively close to the numerical threshold.

## II. AN EFFECTIVE NUMERICAL IDENTIFICATION MEASURE

In this section, we give the detailed description of the simulation of SIR model, propose the numerical identification measure of the epidemic threshold, and make deep analysis of the effectiveness of the numerical measure. In the SIR model, each node can be one of three states which are the susceptible state, infected state, and recovered state, respectively. At the beginning, one node is randomly selected as the initial infected (i.e., seed), and all other nodes are susceptible. At each time step *t*, each susceptible node *i* becomes infected with probability $1-{\left(1-\beta \right)}^{n_{i}}$ if it has one or more infected neighbors, where *n_i_* is the number of its infected neighbors. At the same time, all infected nodes recover (or die) at rate *μ* and the recovered nodes acquire permanent immunity. Time increases by Δt=1, and the dynamical process terminates when all infected nodes are recovered. In this paper, *μ* is set to 1, unless otherwise specified.

### A. Proposing the numerical identification measure

**FIG. 1.**
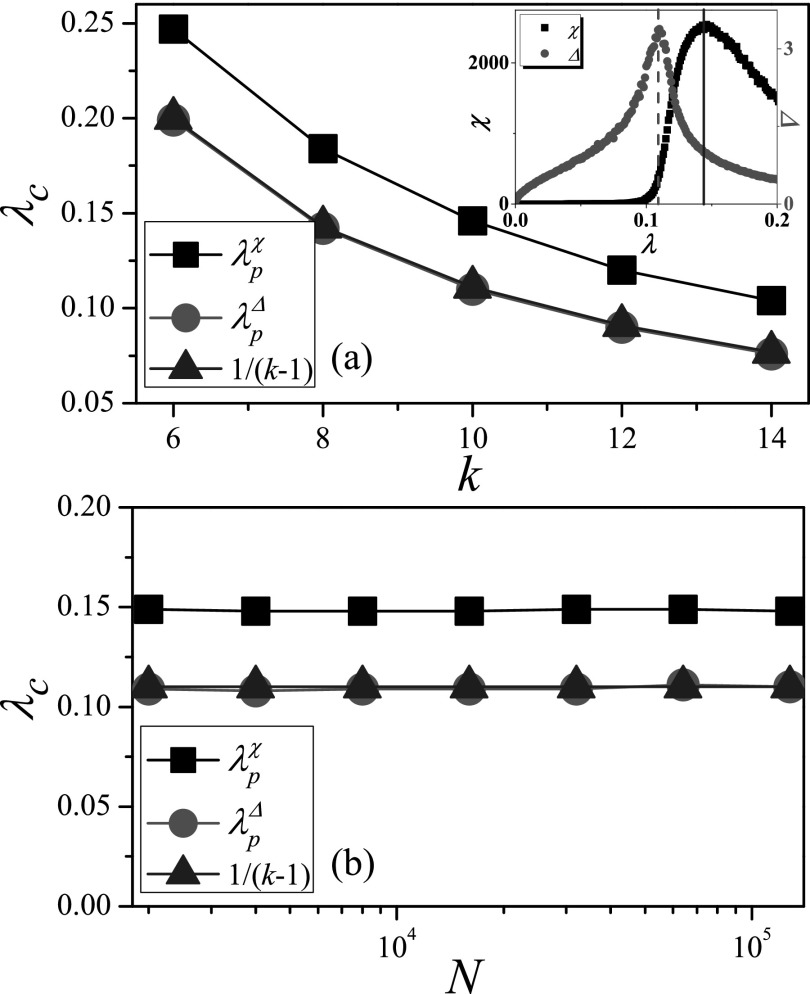
Comparison of theoretical thresholds with numerical thresholds on RRN. (a) The threshold *λ_c_* vs. *k*, where *N* is set to 10^4^. (b) The threshold *λ_c_* vs. *N*, where *k* is set to 10. “Squares,” “circles,” and “triangleups” denote $\lambda_{p}^{\chi }$, $\lambda_{p}^{\Delta }$, and 1/(k−1), respectively. Inset: Susceptibility *χ* and variability Δ as a function of *λ*. The results are averaged over $10^{2}\times 10^{6}$ independent realizations on 10^2^ networks.

The susceptibility measure can not only identify an effective SIS epidemic threshold,^18^ but can also be used to determine the critical point of the percolation process.^26^ Since the connection between SIR and bond percolation is made through the assimilation of the size of the percolating giant component with the final number of recovered individuals,^21^ we check the effectiveness of susceptibility measure *χ* for the SIR model on RRN, where all nodes have exactly the same degree *k*. On these networks, the HMF prediction $\lambda_{c}^{HMF}=1/(k-1)$ (Ref. 5) is accurate for the SIR model. We compare the HMF prediction with the numerical threshold identified by the susceptibility measure $\lambda_{p}^{\chi }$ in Fig. 1(a), where the result shows that the numerical threshold of the SIR model identified by *χ* is significantly larger than 1/(k−1).

Considering that the fluctuation of the outbreak size is large near the epidemic threshold, we try to identify the epidemic threshold by the variability measure^24,25^

$$\Delta =\frac{\sqrt{〈\rho^{2}〉-{〈\rho 〉}^{2}}}{〈\rho 〉},$$ (5)

which is a standard measure to determine the critical point in equilibrium phase on magnetic system.^27^ The inset of Fig. 1(a) shows that the variability Δ exhibits a peak over a wide range of *λ*, so we estimate the epidemic threshold from the position of the peak of the variability $\lambda_{p}^{\Delta }$. On RRN with different values of *k*, we find that $\lambda_{p}^{\Delta }$ is always consistent with the HMF prediction. When the degree *k* is given, we further consider the relationship between the epidemic threshold and the network size *N* in Fig. 1(b), where the numerical thresholds $\lambda_{p}^{\chi }$ and $\lambda_{p}^{\Delta }$ do not change with *N*. $\lambda_{p}^{\Delta }$ is very close to the HMF prediction, while there is an obvious gap between $\lambda_{p}^{\chi }$ and HMF prediction. From the above, we know that the variability Δ can identify an effective SIR epidemic threshold, while the epidemic threshold identified by the susceptibility *χ* is overestimated on RRN. Thus, a new problem has arisen: Why the variability Δ performs well but the susceptibility *χ* goes awry for the SIR model?

### B. Analysis of the effectiveness of numerical identification measure

**FIG. 2.**
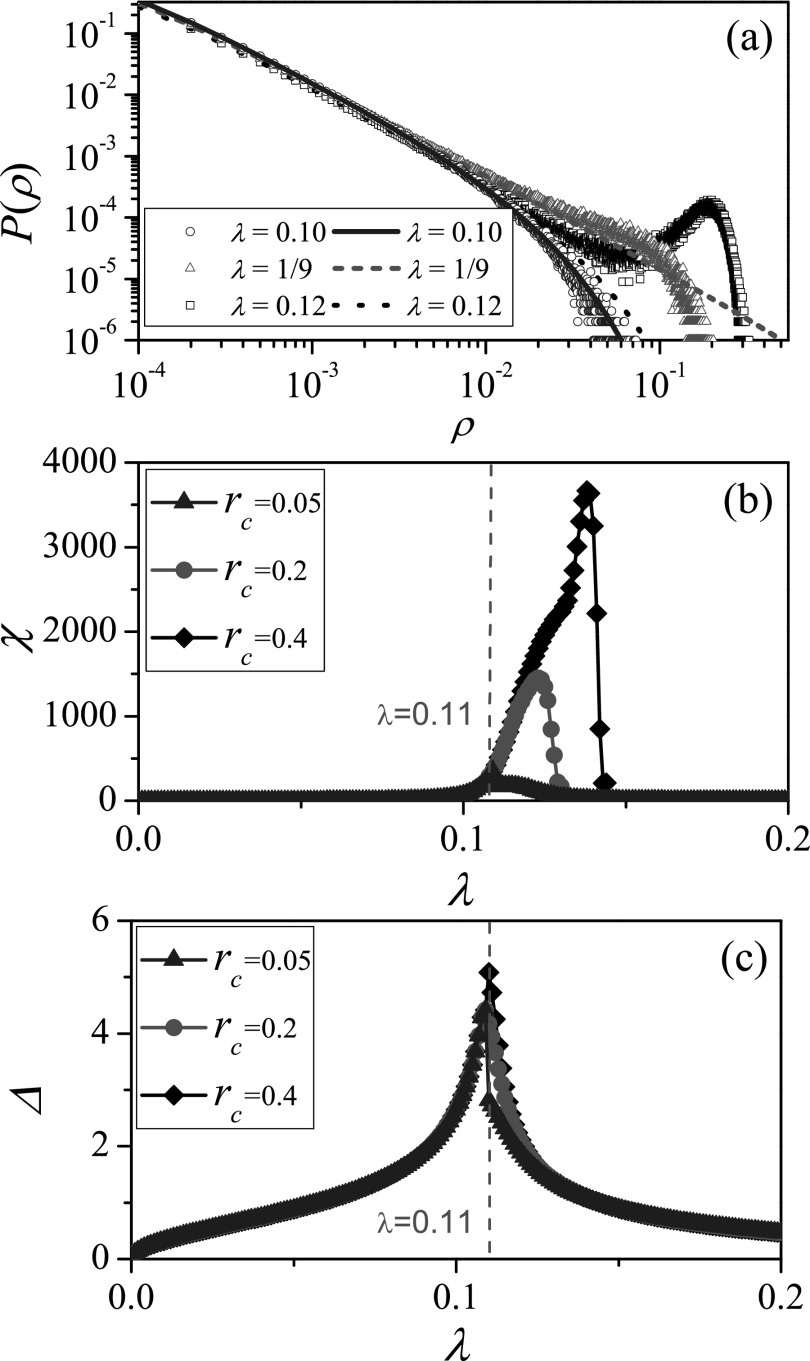
Analysis of effectiveness and robustness of numerical methods on RRN. (a) Numerical distribution of outbreak sizes in SIR model for λ=0.10 (circles), λ=1/9 (triangles), and λ=0.12 (squares), where blue solid, red short dash, and black dot lines represent the theoretical distributions given by Eq. (A4). (b) *χ* vs. *λ*, where only the small outbreak sizes with $\rho \leq r_{c}$ are considered to numerically count *χ*. (c) Δ vs. *λ*, where the lump is assumed to be located at *r_c_* in the theoretical distribution diagram when $\lambda >\lambda_{c}$. In (b) and (c), “triangles,” “circles,” and “diamonds” denote cutoff values *r_c_* = 0.05, 0.2 and 0.4, respectively. The parameters are chosen as $N=10^{4}$ and *k* = 10. The results are averaged over $10^{2}\times 10^{6}$ independent realizations on 10^2^ networks.

Next, we make an analysis of the effectiveness of the numerical identification measures above, by investigating the distribution of outbreak sizes which has the strong heterogeneity near the epidemic threshold. On RRN with *k* = 10, where the epidemic threshold $\lambda_{c}=1/(k-1)=1/9$, Fig. 2(a) shows the distribution of outbreak sizes near *λ_c_*. The outbreak sizes follow approximately an exponential distribution at λ=0.1 that is smaller than *λ_c_*. At $\lambda =\lambda_{c}$, the outbreak sizes follow a power-law distribution $P(\rho )∼\rho^{\alpha }$ with a cutoff at some values, where α≃−1.5.^28–30^ Since the disease may die out quickly or infect a subset of nodes when $\lambda >\lambda_{c}$, the distribution of outbreak sizes is bimodal,^31,32^ with two peaks occurring at ρ=1/N and ρ≃0.2 for λ=0.12, respectively.

Moreover, the theoretical distribution of outbreak sizes (see Appendix for details) is compared with the results obtained by numerical simulations in Fig. 2(a), where the theoretical probability from Eq. (A4) is consistent with the numerical results for relatively small outbreak sizes (ρ<0.05). At the epidemic threshold, the theoretical results also show that the outbreak sizes obey a power-law distribution with the exponent of about −1.5. When $\lambda >\lambda_{c}$, some large outbreak sizes constitute a lump in the numerical scattergram, but the probability of large outbreak sizes cannot be solved from Eq. (A4). We thus speculate that the non-ignorable lump may affect the numerical identification of SIR epidemic threshold for the susceptibility measure.

To verify the speculation, Fig. 2(b) investigates the effectiveness of the susceptibility measures under some cutoff hypotheses. We set the cutoff value of the outbreak size as *r_c_*, which means that only the outbreak sizes with $\rho \leq r_{c}$ are used to numerically count the susceptibility *χ* under the cutoff hypothesis. Three kinds of *r_c_* are considered, where $r_{c}=0.05$ corresponds to the maximum value of small outbreak size before the lump appears in the numerical scattergram, $r_{c}=0.2$ means that the numerical scattergram consists of a part of the lump, and $r_{c}=0.4$ means that there is a complete lump in the numerical scattergram. As shown in Fig. 2(b), the susceptibility measure can indeed give a quite effective estimate of the SIR epidemic threshold when the whole lump is ignored (i.e., $r_{c}=0.05$). With the increase of *r_c_*, the position of peak value of susceptibility *χ* gradually shifts to the right for large outbreak sizes are considered. This shows that the susceptibility *χ* loses its effectiveness in identifying the SIR epidemic threshold due to the existence of the lump.

In Fig. 2(c), the robustness of the variability Δ is further checked in theory. As the numerical distribution of the large outbreak sizes is concentrated, we assume that there is a lump located at *r_c_* with $P(r_{c})=1-\Sigma_{\rho <r_{c}}P(\rho )$ in the theoretical probability distribution diagram of outbreak sizes while $\lambda >\lambda_{c}$. Based on such theoretical distribution, we plot the variability measure as a function of *λ* for different values of *r_c_* in Fig. 2(c). Since the variability Δ measures the heterogeneity of the outbreak size distribution, which is strongest at the epidemic threshold,^28–30^ the peak position of the variability measure does not change with the position of the lump.

From the above analysis, we can conclude that the variability Δ is effective in identifying the epidemic threshold of SIR model, while the bimodal distribution of outbreak sizes for $\lambda >\lambda_{c}$ leads to the overestimation of the SIR epidemic threshold when using the susceptibility *χ*.

## III. VERIFICATION OF THE THEORETICAL PREDICTIONS ON SCALE-FREE AND REAL NETWORKS

We further verify the theoretical predictions on scale-free and real networks, by comparing them with the numerical thresholds from the variability Δ.

### A. Scale-free networks

**FIG. 3.**
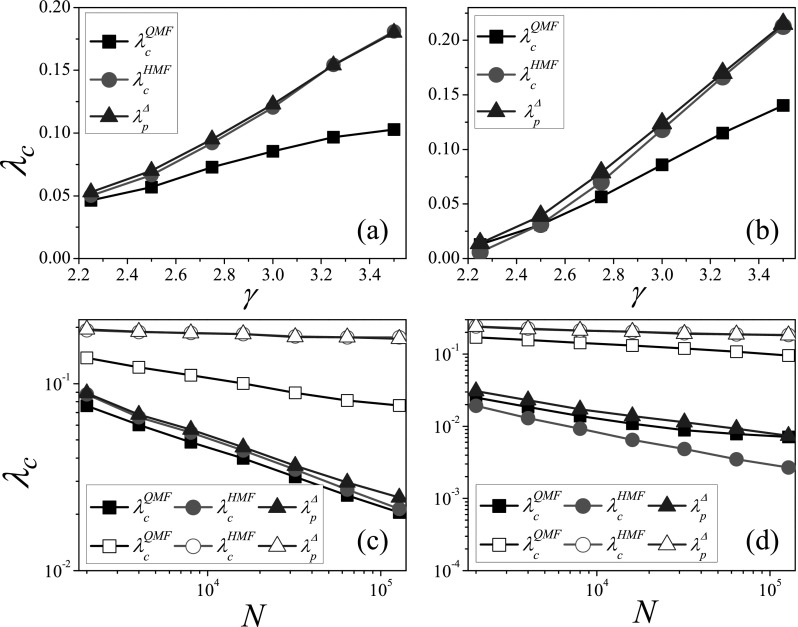
Comparison of theoretical thresholds with numerical thresholds on SFN. The threshold *λ_c_* vs. *γ* on SFN with structural cutoff (a) and natural cutoff (b), where *N* is set to 10^4^. *λ_c_* vs. *N* on SFN with structural cutoff (c) and natural cutoff (d), where solid and empty symbols denote γ=2.25 and 3.50, respectively. “Squares,” “circles,” and “triangles” denote $\lambda_{c}^{QMF},\;\lambda_{c}^{HMF}$, and $\lambda_{p}^{\Delta }$, respectively. The results are averaged over $10^{2}\times 10^{6}$ independent realizations on 10^2^ networks.

We build scale-free networks (SFN) with degree distribution $P(k)∼k^{-\gamma }$ based on the configuration model.^9^ The so-called structural cutoff $k_{max}∼N^{1/2}$ and natural cutoff $k_{max}∼N^{1/\gamma -1}$ (Ref. 33) are considered to constrain the maximum possible degree *k_max_* on SFN. Differently, the degree-degree correlations vanish on scale-free networks with structural off, while the disassortative degree-degree correlations exist when γ<3 for scale-free networks with natural cutoff,^33^ because high degree vertices connect preferably to low degree ones in this case. We consider the SIR model on SFN with structural cutoff in Figs. 3(a) and 3(c), where the SIR epidemic threshold increases monotonically with the degree exponent *γ*, and the variation of epidemic threshold with network size *N* is approximately linear in logarithmic relationship.^16^ The HMF prediction $\lambda_{c}^{HMF}$ is very close to the numerical threshold $\lambda_{p}^{\Delta }$, while there is an obvious difference between the QMF prediction $\lambda_{c}^{QMF}$ and $\lambda_{p}^{\Delta }$.

The SFN with natural cutoff are considered in Figs. 3(b) and 3(d), where the variations of epidemic threshold with *γ* and *N* are similar to the results on SFN with structural cutoff. When γ>3, the HMF prediction is close to the numerical threshold, while there is a gap between the QMF prediction and the numerical threshold. Since the disassortative degree-degree correlations exist for γ<3, there is a slight difference between $\lambda_{c}^{HMF}$ and $\lambda_{p}^{\Delta }$. In Fig. 3(d), the distinction between $\lambda_{c}^{HMF}$ and $\lambda_{p}^{\Delta }$ becomes large with the increase of *N* for γ=2.25, while in such case the QMF prediction is always close to the numerical threshold since the principle eigenvector is delocalized when 2<γ≤5/2.^34^ It can been seen from the above analysis that the numerical method presented provides quantitive indexes for the observations in Ref. 22, where the HMF theory is relatively accurate for the SIR model.

### B. Real-world networks

**FIG. 4.**
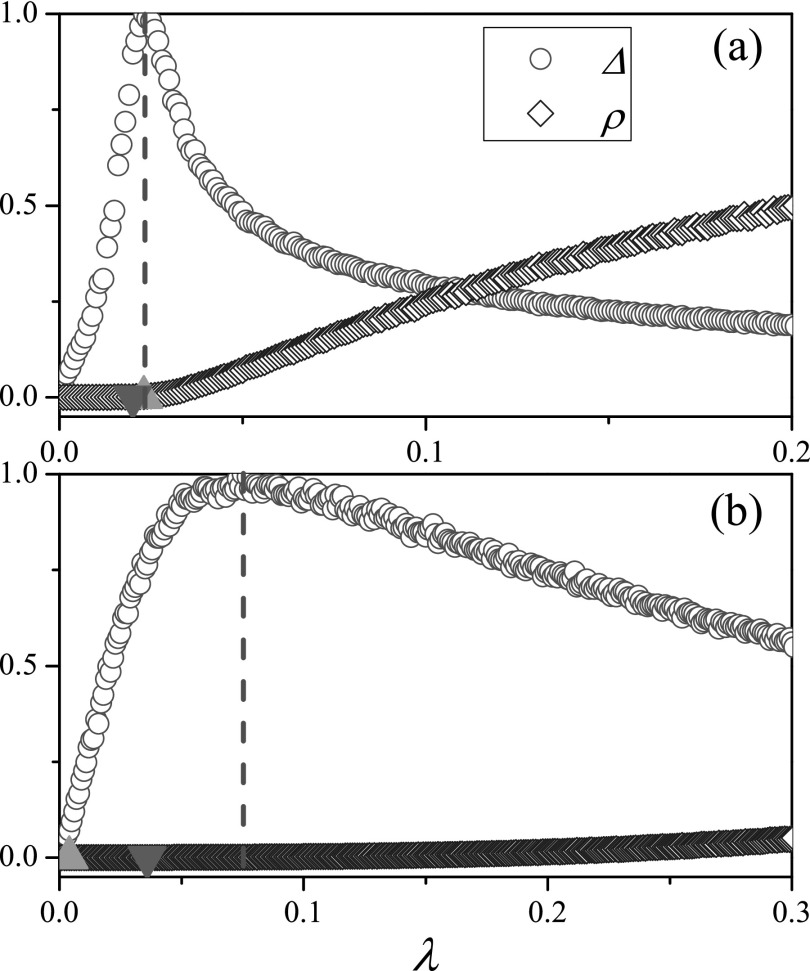
Variability Δ and outbreak size *ρ* as a function of *λ* on Hamsterster full network (a) and Facebook (NIPS) network (b). “Triangleup” and “triangledown” denote $\lambda_{c}^{HMF}=〈k〉/[〈k^{2}〉-〈k〉]$ and $\lambda_{c}^{QMF}=1/\Lambda_{N}$, respectively. The variability Δ for each *λ* is normalized by the maximal variability Δ_*max*_. The results are averaged over 10^6^ independent realizations on each network.

To check the performance of the variability Δ on real-world networks,^35–42^ Fig. 4 depicts Δ as a function of *λ* for Hamsterster full network,^35^ which contains friendships and family links between users of the website hamsterster.com, and Facebook (NIPS) network,^41^ which contains Facebook user-user friendships. The numerical results intuitively show that the variability Δ always reaches a maximum value near the value of *λ* above which the outbreak size *ρ* becomes finite. The theoretical predictions of the HMF theory and of the QMF theory are quite close to the numerical threshold identified by Δ on Hamsterster full network, but they become poor on Facebook (NIPS) network. Considering the difference in the behaviors of the theoretical predictions on the two networks above, the detailed comparisons between the numerical and theoretical thresholds on other real networks are presented in Table I. The results indicate that although the HMF prediction and the numerical threshold $\lambda_{p}^{\Delta }(\text{SI}R)$ are nearly the same for assortative networks, there is an obvious difference between them for the networks showing significant disassortative mixing. The QMF prediction is relatively worse than the HMF prediction for assortative networks, but the former is close to $\lambda_{p}^{\Delta }(\text{SI}R)$ for some disassortative networks (e.g., Router views,^37^ CAIDI,^37^ and email contacts^42^). These findings numerically show the accuracies of existing theoretical predictions on real-world networks.

## IV. CONCLUSION AND DISCUSSION

In summary, we have studied the numerical identification of SIR epidemic threshold on complex networks with finite size. We have checked the effectiveness of the susceptibility measure for SIR on RRN, where the HMF is exact. The results showed an obvious gap between the numerical threshold identified by the susceptibility *χ* and the HMF prediction. Then, we proposed the numerical identification method by analyzing the peak of the epidemic variability, and found that the numerical threshold identified by the variability measure Δ agrees very well with the HMF prediction on RRN.

In order to get a deep understanding of the effectiveness of the two numerical measures above, we have analyzed the distribution of outbreak sizes near the epidemic threshold *λ_c_*. The outbreak sizes follow approximately an exponential distribution when $\lambda <\lambda_{c}$. At the epidemic threshold, the outbreak sizes follow a power-law distribution with the exponent of about −1.5. When $\lambda >\lambda_{c}$, the numerical distribution of outbreak sizes is bimodal with two peaks occurring at ρ=1/N and *O*(1), respectively. The probability of small outbreak sizes in theory is consistent with that obtained by numerical simulations, but the probability of large outbreak sizes that constitute a lump in the numerical scattergram cannot be obtained theoretically. Based on the analysis of the cutoff hypothesis of the outbreak size distribution, we found that the susceptibility measure can give a fairly effective SIR epidemic threshold when the lump is ignored. Since the variability measure reflects the heterogeneity of the outbreak size distribution, it is always effective in identifying the epidemic threshold, where the distribution of outbreak sizes has the very strong heterogeneity.

We further employed the variability measure to verify the theoretical predictions on scale-free and real networks. The HMF prediction is close to the numerical threshold on most of the networks, but on SFN with natural cutoff and degree exponent γ<5/2, it becomes poor due to the existence of disassortative mixing. Similarly, the HMF prediction agrees well with the numerical method on real networks with assortative mixing, while it becomes very poor for disassortative networks, where the QMF prediction is relatively close to the numerical threshold. These findings provide quantitive indexes for the accuracies of existing theoretical predictions from the perspective of simulation.

**FIG. 5.**
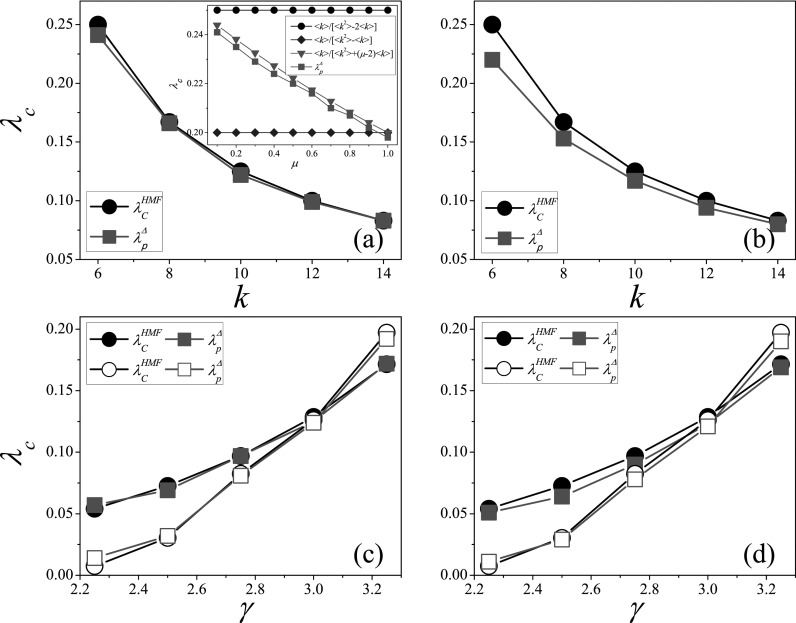
Comparison of theoretical thresholds with numerical thresholds for μ<1. The threshold *λ_c_* vs. *k* for μ=0.1 (a) and μ=0.5 (b) on RRN. The threshold *λ_c_* vs. *γ* for μ=0.1 (c) and μ=0.5 (d) on SFN, where solid and empty symbols denote SFN with structural cutoff $k_{max}∼N^{1/2}$ and natural cutoff $k_{max}∼N^{1/\gamma -1}$, respectively. “Circles” and “squares” denote $\lambda_{c}^{HMF}$ and $\lambda_{p}^{\Delta }$, respectively. Inset: *λ_c_* as a function of *μ* on RRN with $N=10^{4}$ and *k* = 6. The results are averaged over $10^{2}\times 10^{6}$ independent realizations on 10^2^ networks.

As part of the discussion, we have considered the epidemic threshold for μ<1 in Fig. 5. The results on RRN and SFN all show that the numerical thresholds for μ=0.1 are a little larger than those for μ=0.5. As shown in the inset, μ→0 leads to an epidemic threshold close to Eq. (4), while μ→1 leads to an epidemic threshold close to Eq. (3). It should be pointed out that the numerical threshold of SIR model is inclined to the theoretical prediction $\lambda_{c}=\frac{〈k〉}{〈k^{2}〉+(\mu -2)〈k〉}$ from the edge-based compartmental theory^43,44^ when 0<μ<1. These findings could be complementary to some existing results.^4^

**FIG. 6.**
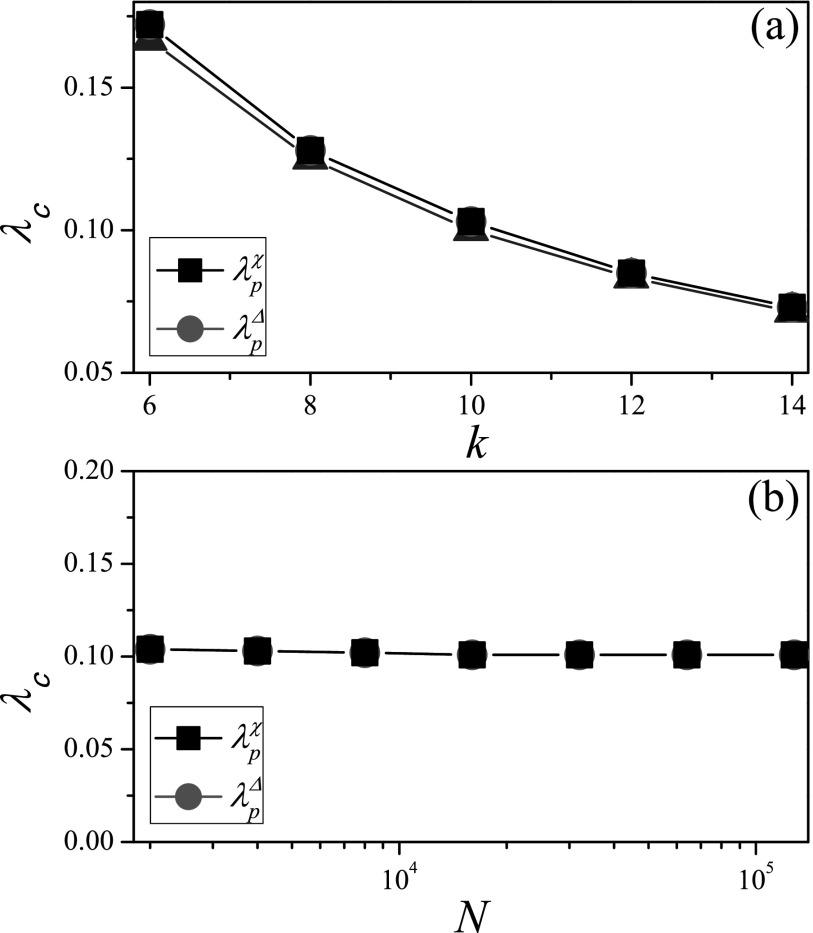
Comparison of theoretical thresholds with numerical thresholds for SIS model on RRN. (a) The threshold *λ_c_* vs. *k*, where *N* is set to 10^4^. (b) The threshold *λ_c_* vs. *N*, where *k* is set to 10. “Squares” and “circles” denote $\lambda_{p}^{\chi }$ and $\lambda_{p}^{\Delta }$, respectively. The results are averaged over $10^{2}\times 10^{6}$ independent realizations on 10^2^ networks.

Moreover, we have tried applying the variability measure to the identification of the SIS epidemic threshold. As shown in Fig. 6 and Table I, the numerical threshold $\lambda_{p}^{\Delta }$ from the variability measure agrees very well with the threshold $\lambda_{p}^{\chi }$ identified by the susceptibility measure, whose validity for the SIS model has been confirmed in Ref. 18. This shows that the variability measure can also provide an effective estimate of the SIS epidemic threshold.

We have put forward a numerical method for identifying the epidemic threshold for SIR model, which is also suitable for the SIS model. This method can effectively identify epidemic thresholds on various networks, and could be extended to other dynamical processes such as information diffusion and behavior spreading. Further work should be done to check the effectiveness of this method on more complicated networks (e.g., temporal networks^45^ and multilayer networks^46^). Besides, the accurate analytic approximation of the epidemic threshold for general networks remains an important problem. This work helps to verify theoretical analysis of epidemic threshold and would promote further studies on phase transition of epidemic dynamics.

## Figures and Tables

**TABLE I.** Characteristics and epidemic thresholds of real-world networks. *N* is the network size, *k_max_* is the maximum degree, *r* is the correlation coefficient of the degrees, $\lambda_{c}^{HMF}$ and $\lambda_{c}^{QMF}$ are the HMF and QMF predictions for SIR, respectively, $\lambda_{p}^{\Delta }$(SIR) denotes the numerical threshold of SIR identified by Δ, and $\lambda_{p}^{\Delta }$(SIS) and $\lambda_{p}^{\chi }$(SIS) represent the numerical thresholds of SIS identified by Δ and *χ*, respectively.

Network	*N*	*k_max_*	*r*	$\lambda_{c}^{HMF}$	$\lambda_{c}^{QMF}$	$\lambda_{p}^{\Delta }$(SIR)	$\lambda_{p}^{\Delta }$(SIS)	$\lambda_{p}^{\chi }$(SIS)
Hamsterster full^35^	2000	273	0.023	0.023	0.020	0.023	0.025	0.025
Brightkite^36^	56739	1134	0.010	0.016	0.010	0.014	0.012	0.012
arXiv astro-ph^37^	17903	504	0.201	0.015	0.011	0.012	0.012	0.012
Pretty good privacy^38^	10680	206	0.239	0.056	0.024	0.053	0.033	0.033
US power grid^39^	4941	19	0.003	0.348	0.134	0.446	0.261	0.264
Euroroad^40^	1039	10	0.090	0.479	0.249	0.498	0.331	0.331
Facebook (NIPS)^41^	2888	769	−0.668	0.004	0.036	0.075	0.079	0.077
Route views^37^	6474	1458	−0.182	0.006	0.022	0.037	0.034	0.036
CAIDA^37^	26475	2628	−0.195	0.004	0.014	0.019	0.019	0.019
email contacts^42^	12625	576	−0.387	0.009	0.02	0.027	0.024	0.025

## APPENDIX: THE THEORETICAL DISTRIBUTION OF SMALL OUTBREAK SIZES

For the case of the SIR model and similar models with no steady-state, the static properties (e.g., the final outbreak size and the epidemic threshold) of the epidemic outbreak can be mapped into a suitable bond percolation problem. In this framework, the distribution of occupied cluster sizes is related to the distribution of outbreak sizes. To get the distribution of small outbreak sizes in the SIR model with a fixed value of *λ* when recovery rate *μ* = 1, we will present the derivation of the distribution of small occupied cluster sizes in bond percolation with bond occupation probability *λ*.^21^

After the percolation process on a general network with arbitrary degree distribution *p_k_*, the average degree of the occupied network *A*_1_, which composes of vertices and occupied edges, is $〈k_{T}〉=\lambda 〈k〉$, where 〈k〉 is the average degree of the original network *A*_0_. And, the size distribution of the small subgraphs of network *A*_1_ is

$$\pi_{s}=\frac{〈k_{T}〉}{(s-1)!}{\left[\frac{d^{s-2}}{dz^{s-2}}{\left[g_{1}(z)\right]}^{s}\right]}_{z=0},$$ (A1)

where *s* is the small subgraphs size and $g_{1}(z)$ is the generating function of the excess degree of network *A*_1_. In addition, the generating function of degree distribution of *A*_1_ is

$$g_{0}(z)=\sum_{k=0}^{\infty }p_{k}{\left(1-\lambda +z\lambda \right)}^{k},$$

and we thus have

$$g_{1}(z)=\frac{g_{0}^{\prime }(z)}{g_{0}^{\prime }(1)}.$$

In a random regular network, which has an unique degree *k* with *p_k_* = 1, we can easily obtain that

$$g_{0}(z)={\left[1+(z-1)\lambda \right]}^{k},$$ (A2)

and

$$g_{1}(z)={\left[1+(z-1)\lambda \right]}^{k-1}.$$ (A3)

Substituting Eq. (A3) into Eq. (A1), we can obtain the distribution of small outbreak sizes

$$\pi_{s}=\frac{k\Gamma (a_{2})}{\Gamma (a_{0})\Gamma (a_{1})}\;\lambda^{s-1}{\left(1-\lambda \right)}^{s(k-1)-(s-2)},$$ (A4)

where $\Gamma (x+1)=x!,a_{0}=(s-2),a_{1}=s(k-1)-(s-1)$, and $a_{2}=s(k-1)-1$.
